# Carotid endarterectomy versus conservative management of the asymptomatic carotid stenosis before coronary artery bypass grafting: a retrospective study

**DOI:** 10.1186/s12872-020-01585-z

**Published:** 2020-06-19

**Authors:** Mario Lescan, Mateja Andic, Oana Bartos, Christian Schlensak, Migdat Mustafi

**Affiliations:** Department of Thoracic and Cardiovascular Surgery, University Medical Center Tübingen, Hoppe-Seyler Strasse 3, D-72076 Tübingen, Germany

**Keywords:** Carotid endarterectomy, Cardiac surgery, Carotid stenosis, Staged procedure, Simultaneous procedure

## Abstract

**Background:**

Our retrospective single-center study aimed to evaluate the safety of the carotid endarterectomy (CEA) in comparison to patients with untreated asymptomatic carotid stenosis ≥60% before CABG.

**Methods:**

This single-center retrospective study included 174 patients with asymptomatic unilateral carotid stenosis treated between 2004 and 2017 with CABG. Thereof 106 patients had CEA before cardiac surgery either by a simultaneous (*n* = 62) or staged (*n* = 44) approach. Patients with untreated carotid stenosis served as control (no-CEA group; *n* = 68).

**Results:**

The mean stenosis grade was higher in the CEA group (CEA 83% (±1), no-CEA 71% (±1) *p* < 0.0001). The overall stroke rate was 5/174 (3%) and was due to a high incidence of stroke in the no-CEA group (CEA: 0/106 (0%); No-CEA 5/68 (7%) *p* = 0.0083). The overall mortality was 1% and comparable between the groups (CEA: 2/106 (2%); No-CEA 0/68 (0%) *p* = 0.5211). Stroke related mortality was not observed. The groups were similar regarding the incidence of myocardial infarction (*p* = 1.0), atrial fibrillation (*p* = 0.1931), delirium (*p* = 0.2106) and IMC/ICU stay (*p* = 0.1542). No significant difference in the subgroup analysis was found between the simultaneous and staged approach regarding the myocardial infarction (*simultaneous*: 1/62 (1%); *staged:* 1/44 (1%); *p* = 1.0).

**Conclusions:**

CEA performed as a staged procedure in local anesthesia or a simultaneous procedure in general anesthesia, may reduce the stroke risk prior to CABG.

## Background

The occurrence of stroke during cardiac surgery is a complication associated with a poor in-hospital and long-term prognosis [1]. The incidence of carotid artery stenosis concurrent with coronary artery disease varies between 2 and 22% and it is preoperatively found in up to 8% of patients undergoing CABG [2–5]. The treatment of the asymptomatic carotid stenosis before CABG was shown to reduce stroke [6]. However, the guidelines recommend carotid revascularization prior to cardiac surgery only for symptomatic and bilateral carotid stenosis > 70–80% [7, 8]. The cut-off stenosis grade justifying the CEA indication for the asymptomatic, unilateral stenosis, and the ideal timing of the carotid revascularization (staged or simultaneous with CABG) are not well established. Our study aimed to compare the outcomes of patients with unilateral asymptomatic carotid stenosis ≥60%, who received CEA before CABG as a simultaneous or a staged procedure, to those with untreated carotid stenosis.

## Methods

### Institutional protocol

All patients referred to CABG in our institution were screened for carotid artery stenosis with duplex sonography. Our center’s protocol included the treatment of an asymptomatic carotid artery stenosis before CABG at a stenosis grade ≥ 70% and depending on the assessment of the attending surgeon. Thus, in the time period between 2004 and 2016, 15 asymptomatic patients with a 60–69% stenosis, 40 patients with a 70–79% stenosis and 13 patients with a stenosis grade ≥ 80% have not been treated by CEA before CABG and served as a control group in this study (no-CEA group). The cut-off stenosis value in the control group was chosen according to the national and international guidelines for isolated carotid artery disease, which recommend the treatment of asymptomatic stenosis ≥60% [9, 10].

In the past years, two different revascularization strategies were pursued in the management of unilateral asymptomatic carotid artery stenosis prior to CABG procedures. From 2004 to 2012 simultaneous (CEA + CABG) procedure in general anesthesia was performed, and since 2012 staged approach in local anesthesia was favorized. Patients with unilateral carotid stenosis ≥60% and isolated CABG were identified throughout the study period and mainly between 2010 and 2014 (45; 66%).

### Staged procedure

During the same hospital stay CEA was performed before CABG under local anesthesia (with ultrasound-guided combined superficial and deep cervical plexus block) and near-infrared spectroscopy surveillance (INVOS™, Medtronic, Minneapolis, U.S.A.). Carotid shunting was performed only in patients with obvious clamping ischemia (selective shunting). The carotid reconstruction was performed either as a patch thrombendarterectomy or eversion endarterectomy. Postoperatively, all patients were neurologically examined by the anesthesiologists and the surgical team. The success of the carotid revascularization was additionally evaluated by duplex sonography performed by the neurologists before discharge in the simultaneous CEA group and before CABG in the staged CEA group. The CABG procedure was performed after 4 days in median.

### Simultaneous procedure

The CEA was performed simultaneously with cardiac surgery under general anesthesia before the initiation of cardiopulmonary bypass. Routine shunting was conducted and neuro-monitoring was performed by near-infrared spectroscopy (INVOS™, Medtronic, Minneapolis, U.S.A.) during the entire procedure. The carotid reconstruction was performed as a patch thrombendarteriectomy. The patients were neurologically evaluated after extubation by the intensive care physicians and the surgical team.

### Data acquisition

Data were acquired from the medical records: 1219 CEA procedures were performed from 2004 to 2017 at our center, while CEA and CABG were conducted in 206 cases during the same hospital stay.

Exclusion criteria were contralateral stenoses ≥60% or contralateral carotid occlusion (*n* = 31) and symptomatic carotid stenosis with neurological events within 6 months before the operation (*n* = 30). Preoperative hemodynamic instability requiring emergency interventions (IABP, ECLS) and patients with preoperatively elevated cardiac enzymes led to the exclusion from the analysis (*n* = 39). Ultimately, 106 patients with CEA + CABG were included in the analysis (CEA group).

### Study parameters

The demographic data and important risk factors for perioperative morbidity and mortality were compared between the study groups: gender, age, symptomatic carotid stenosis, ejection fraction, hypertension, renal insufficiency requiring dialysis, nicotine abuse, dyslipoproteinemia, and diabetes mellitus. Indicators of perioperative morbidity and mortality were the following: in-hospital mortality, in-hospital ipsilateral stroke, in-hospital ipsilateral TIA, any ipsilateral neurological event (stroke+TIA), carotid-related mortality, and the length of the intensive care unit/ intermediate care (ICU/IMC) stay.

Postoperative myocardial infarction was defined as the increase of creatine kinase levels (> 800 U/l) with a concurrent significant CK-MB increase and the ST-segment elevation in the ECG [11]. The severity of postoperative stroke was assessed with the NIHSS score in the acute phase and the modified Rankin scale (mRS) at discharge [11].

### Statistical analysis

The SPSS 23 software package (IBM Corporation, Somer, NY, USA) was used to perform the statistical analysis. Normality testing was assessed with the Kolmogorow-Smirnow test. Normal data were further analyzed with the two-tailed t-tests and categorical variables were processed by Fisher’s exact test. Numerical and categorical variables are presented as the mean (±standard error) and percentages, respectively. *P* values < 0.05 were considered significant.

## Results

Overall 174 patients with an asymptomatic carotid artery stenosis ≥60% were referred to CABG procedure in our institution and included in the study. In the CEA group (*n* = 106), the carotid revascularization was performed as staged (*n* = 44) or synchronous (*n* = 62) procedure. In the no-CEA group, 68 individuals with a stenosis grade ≥ 60% were referred to CABG without carotid revascularization and served as a control group.

### Demographic data

Both study groups were comparable in terms of their demographic data and risk factors (Table 1.). Male individuals were treated more often in both study groups, whereas the percentage of treated women was higher in the CEA group (CEA: 34/106 (32%); No-CEA 10/68 (14%); *p* = 0.0121; Table 1.). Patients from the no-CEA group had more often dyslipoproteinemia (CEA: 37/106 (35%); No-CEA 35/68 (51%); *p* = 0.0401; Table 1.). The mean carotid stenosis grade was significantly higher in the CEA group (CEA: 83% (±1); No-CEA 71% (±1) *p* < 0.0001; Table 1.).

**Table 1:** Demographic and operative parameters of the CEA and no-CEA study groups

Characteristic	CEA^**1**^	No-CEA	p
Female sex	34 (32%)	10 (14%)	0.0121*
Age (years)	71 (±1)	71 (±1)	0.7101
Diabetes mellitus	46 (43%)	31 (46%)	0.8758
Dyslipoproteinemia	37 (35%)	35 (51%)	0.0401*
Arterial hypertension	15 (14%)	11 (16%)	0.8280
Nicotine abuse			
Current smoker	7 (7%)	8 (12%)	0.0963
Prior smoker	23 (22%)	22 (32%)	
No smoker	76 (72%)	38 (56%)	
History of stroke	16 (15%)	10 (15%)	0.9441
Peripheral vascular disease	19 (18%)	17 (25%)	0.3376
Preoperative dialysis	3 (3%)	1 (1%)	1.0
Ejection fraction (%)	51 (±1)	50 (±2)	0.4129
Grade of carotid stenosis (%)	83 (±1)	71 (±1)	< 0.0001*
Preoperative patient status			
elective	81 (76%)	46 (68%)	0.2037
urgent	25 (24%)	22 (32%)	
OPCAB	18 (17%)	15 (22%)	0.4324

Ordinal parameters are reported as percentage n (%). Continuous variables are presented as means (±standard error)

^1^CEA carotid endarterectomy

*statistically significant

### Outcome

The overall stroke rate was 3%, whereby strokes were not observed in the CEA group. The incidence of ipsilateral stroke was 7% in the no-CEA group (CEA: 0/106 (0%); No-CEA 5/68 (7%) *p* = 0.0083; Table 2.). TIA occurred in one patient from the no-CEA group, which resulted in a total neurological event rate of 9% in this group (*p* = 0.0019). Patients with stroke had an ipsilateral carotid stenosis grade between 60 and 90%: 1 patient with a 60–69% stenosis (1/15; 7%), 2 patients with a 70–79% stenosis (2/40; 5%) and 2 patients with a stenosis ≥80% (2/13; 15%; *p* = 0.4569; Table 3.). Symptom onset was after extubation in 3 out of 5 patients. The initial NIHSS score range was between 5 and 20 points (Table 3.). An initial NIHSS score > 10 was associated with a severe neurological deficit at discharge (mRS; Table 3.). Two patients with postoperative strokes were treated urgently: one patient with carotid artery stenting, the second with a CEA (Table 3.). The remaining patients were treated conservatively. Urgent cardiac operation (*p* = 0.7201) and the history of stroke (*p* = 0.7476) were not associated with a higher stroke incidence.

**Table 2:** Postoperative outcomes of the CEA and no-CEA study groups

Postoperative outcome	CEA^**1**^	No-CEA	p
In-hospital mortality	2 (1.9%)	0 (0%)	0.5211
In-hospital stroke	0 (0%)	5 (7.4%)	0.0083*
In-hospital TIA	0 (0%)	1 (1%)	0.2105
Any neurological event	0	6 (9%)	0.0019*
Myocardial infarction	1 (1%)	1 (1%)	1.0
IMC^2^/ICU^3^ stay	7 (±1)	9 (±1)	0.1542
Resternotomy	5 (5%)	5 (7%)	0.5153
Dysphagia	5 (5%)	8 (12%)	0.1367
Nerve injuries	2 (2%)	1 (1%)	1.0
Delirium	14 (13%)	14 (21%)	0.2106
Atrial fibrillation	20 (19%)	19 (28%)	0.1931

Ordinal parameters are reported as percentage n (%). Continuous variables are presented as means (±standard error)

^1^CEA carotid endarterectomy

^2^IMC intermediate care

^3^ICU intensive care unit

*statistically significant

**Table 3:** Overview of patients with stroke

No.	Stenosis grade	Atrial fibrillation	Symptom onset (days after extubation)	Initial NIHSS^1^ score	Therapy	mRS^2^ score at dis-charge
1	60%	No	0	5	conservative	0
2	70%	No	0	20	conservative	5
3	80%	No	3	7	CAS^3^	2
4	90%	No	2	8	CEA^4^	0
5	70%, exulcerated plaque	No	0	14	conservative	4

^1^NIHSS National Institute of Health Stroke Scale

^2^mRS modified Rankin Score

^3^CAS carotid artery stenting

^4^CEA carotid endarterectomy

Overall mortality was 1% and it was not related to carotid stenosis or CEA: one patient died from a postoperative low cardiac output syndrome, the other patient from sepsis. There was no relevant difference in terms of mortality (CEA: 2/106 (2%); No-CEA 0/68 (0%) *p* = 0.5211; Table 2.) and myocardial infarction (CEA: 1/106 (1%); No-CEA 1/68 (1%) *p* = 1.0; Table 2.) between the groups. Intergroup differences for the incidence of postoperative hematoma, IMC/ICU stay, resternotomy, dysphagia, nerve injuries, delirium, and atrial fibrillation were not significant.

In the subgroup analysis of patients operated with a simultaneous or staged approach, the mortality was comparable (*simultaneous*: 1/62 (1%); *staged:* 1/44 (1%) *p* = 1.0; Table 4.). The other predictors of poor postoperative outcome were comparable and not statistically significant. Particularly, the myocardial infarction was only observed after the simultaneous approach (*simultaneous*: 1/62 (2%); *staged:* 0/44 (0%) *p* = 1.0; Table 4.). No patient from the staged subgroup had a myocardial infarction or required urgent cardiac surgery during the interstage phase (time between carotid and cardiac surgery). Among all patients assigned to the staged group, carotid and cardiac surgery were accomplished during the same hospital stay.

**Table 4:** Postoperative outcomes within the staged CEA and simultaneous CEA subgroups

Postoperative outcome	Staged CEA^**1**^	Simultaneous CEA	p
In-hospital mortality	1 (2%)	1 (2%)	1.0
In-hospital stroke	0 (0%)	0 (0%)	–
Perioperative myocardial infarction	0 (0%)	1 (2%)	1.0
Revision/neck haematoma	0 (0%)	1 (2%)	1.0
IMC^2^/ICU^3^ stay	9 (±2)	5 (±1)	0.4233
Resternotomy	3 (7%)	2 (3%)	0.6470
Dysphagia	2 (5%)	3 (5%)	1.0
Nerve injuries	0 (0%)	2 (3%)	0.5098
Delirium	5 (11%)	9 (15%)	0.7742
Atrial fibrillation	7 (16%)	13 (21%)	0.6179

Ordinal parameters are reported as percentage n (%). Continuous variables are presented as means (±standard error)

^1^CEA carotid endarterectomy

^2^IMC intermediate care

^3^ICU intensive care unit

## Discussion

The most important findings of this study can be summarized as follows:

(i) The treatment of the asymptomatic carotid artery stenosis ≥60% before CABG reduced the incidence of postoperative stroke after CABG in a small retrospective single-center cohort
(ii) Staged approach with CEA in local anesthesia and selective shunting versus simultaneous approach with CEA + CABG in general anesthesia and routine shunting, showed comparable safety prior to CABG.
(iii) Regarding the staged approach, the period between CEA and CABG did not bear an increased risk of cardiac complications, if both operations were performed during the same hospital stay.

### Study design

Previously, different cut-off stenosis grade values for the treatment of asymptomatic carotid stenosis before CABG were reported and different approaches regarding the timing of the CEA prior to CABG were described [6, 7, 12].

The cut-off value (≥60%) used in our study was derived from the national and international guidelines for the treatment of isolated carotid stenosis, which refer to ACAS and ACST-1 trials, and recommend the treatment of an asymptomatic 60–99% stenosis [9, 10, 13, 14]. Thus, in comparison to the prospective studies evaluating the risk and benefit of CEA before CABG the cut-off value in this study was lower (CABACS: 80%; Illuminati et al. 70%) [6, 15].

Our retrospective single-center study aimed to report the in-hospital outcomes of patients with unilateral asymptomatic carotid stenosis who were treated either with CEA + CABG or isolated CABG. The use of synchronous and staged CEA approach in our center in the past and the treatment of a considerable number of patients with isolated CABG in the presence of an untreated unilateral carotid stenosis, who served as a control group (no-CEA), enabled the performance of our study. Thus, the comparison of CEA and no-CEA patients was performed and a subgroup analysis of patients with two different carotid revascularization strategies was feasible. Currently, the evidence for the treatment of asymptomatic carotid stenosis prior to CABG is still limited. The AHA guidelines promote CEA before cardiac surgery in unilateral symptomatic stenosis only in symptomatic patients [7]. In contrast, Illuminati et al. showed the superiority of the CEA over conservative asymptomatic carotid stenosis (≥70%) treatment before cardiac surgery regarding stroke prevention [6]. However, the recent ESC/EACTS guidelines do not recommend the “routine prophylactic carotid revascularization” before CABG (Level of Evidence III, C) although CEA is recommended in case of “one or more characteristics, that may be associated with an increased risk of stroke” (Level of Evidence IIb, C) [16]. The recent multicenter CABACS trial recruited 129 asymptomatic patients with a stenosis grade ≥ 80% and found no benefit of simultaneous CEA [15]. However, the study did not reach the prespecified sample size and was stopped due to insufficient recruitment.

### Stroke

The occurrence of stroke after CABG is multifactorial. Embolic complications including air embolism, thromboembolism, and atheroembolism from the aorta are regarded as the predominant etiological factors for stroke during open heart surgery [17]. Furthermore, the postoperative atrial fibrillation is related to stroke in 5 to 37% of stroke cases after CABG [18, 19].

In the presence of a carotid artery stenosis, hemodynamic instability may lead to a carotid-associated hemodynamic stroke during CABG. Illuminati et al. showed the benefit of CEA prior to CABG in their randomized prospective study [6]. The stroke rate in the CEA group was 1.0%, compared to 8.8% in the group without carotid revascularization. The majority (84%) of their CEA group underwent simultaneous CEA + CABG. Therefore, the difference between the simultaneous and staged procedures could not be well assessed due to the small number of patients treated with the staged approach. Gopaldas et al. showed in a meta-analysis a comparable rate of postoperative stroke in the staged and synchronous CEA groups [12].

In our study, strokes occurred only in the no-CEA group with a stroke rate of 7.4% which is comparable to the findings by Illuminati et al. This supports the idea of stroke risk reduction in patients with asymptomatic carotid stenosis prior to CABG. The percentage of staged CEA in our analysis amounted to 42% and both CEA subgroups (synchronous and staged) showed equivalent capacity to reduce stroke risk before CABG.

### Myocardial infarction

When compared to the studies including patients who underwent CEA and cardiac surgery, the overall rate of myocardial infarction in our study was lower (1.9%), which may be explained by the exclusion of critically ill patients with elevated cardiac enzymes before admission to our center. The interstage phase between the CEA and CABG surgery was previously reported as a vulnerable period for myocardial infarction. Thus, a meta-analysis by Naylor et al. reported the incidence of myocardial infarction of 3.6% for the simultaneously operated group and 6.5% for the staged group [20]. The higher risk of cardiac complications in the staged group was also reported by Gopaldas et al. and other authors [12, 21–24]. Shishebor et al. reported that cardiac events in the interstage phase promote mortality after cardiac surgery [21]. Myocardial infarction in the interstage phase was not observed in our study population. This may be related to our staged strategy, in which the completion of both surgical procedures during one hospital stay was aimed. The interstage phase was kept short (median: 4 days) to prevent the occurrence of cardiac complications in an out-of-hospital setting. Many previously published reports do not specify their interstage time interval. Reported intervals vary from 1.8 to 52 days [25, 26]. Nabagiez et al. promoted earlier cardiac surgery (within 1.8 days) and showed a low cardiac complication rate comparable to our results.

### Limitations

The present study is based on retrospective data and represents a single-center experience with low power: all information was assessed from the data available in the medical records. The main limitation of any study reporting the neurological outcome after CABG is the multifactorial nature of stroke after CABG: embolic strokes are more frequent than carotid-associated strokes, which may bias the results of the study. Furthermore, the significantly lower stenosis grade in the control group may underestimate the stroke risk in comparison to the CEA group.

## Conclusions

CEA may have the potential to reduce the occurrence of perioperative strokes in patients with unilateral asymptomatic carotid stenosis undergoing CABG. The neurological outcomes of patients with a staged or synchronous approach were comparable. The staged procedure allows for optimal neurological monitoring during the CEA in local anesthesia, without increasing the risk of cardiac complications in the interstage phase. However, a prospective three-armed trial is necessary to compare the outcome of CABG patients with asymptomatic stenosis without CEA, staged, or simultaneous CEA approach.

## Data Availability

The datasets used and/or analyzed during the current study are available from the corresponding author on reasonable request.

## Abbreviations

ACAS: Asymptomatic carotid atherosclerosis study
ACST-1: Asymptomatic carotid surgery trial
AHA: American Heart Association
CABG: Coronary artery bypass graft
CABACS: CABG surgery in patients with asymptomatic carotid stenosis
CAS: Carotid artery stenting
CEA: Carotid endarterectomy
CK: Creatine kinase
EACTS: European association for cardio-thoracic surgery
ECG: Electrocardiography
ECLS: Extracorporeal life support
ESC: European society of cardiology
IABP: Intra-aortic balloon pump counterpulsation
ICU: Intensive care unit
IMC: Intermediate care unit
mRS: Modified Rankin scale
NASCET: The north American symptomatic carotid Endarterectomy trial
NIHSS: National Institute of health stroke scale
